# Sake Protein Supplementation Affects Exercise Performance and Biochemical Profiles in Power-Exercise-Trained Mice

**DOI:** 10.3390/nu8020106

**Published:** 2016-02-20

**Authors:** Yi-Ming Chen, Che-Li Lin, Li Wei, Yi-Ju Hsu, Kuan-Neng Chen, Chi-Chang Huang, Chin-Hsung Kao

**Affiliations:** 1 Graduate Institute of Sports Science, National Taiwan Sport University, Taoyuan 33301, Taiwan; 1021302@ntsu.edu.tw (Y.-M.C.); 1031303@ntsu.edu.tw (C.-L.L); 1041302@ntsu.edu.tw (Y.-J.H.); 1040215@ntsu.edu.tw (K.-N.C.); 2 Department of Orthopedic Surgery, Taipei Medical University-Shuang Ho Hospital, New Taipei City 23561, Taiwan; 3 Department of Neurosurgery, Taipei Medical University-WanFang Hospital, Taipei City 11696, Taiwan; nsweili@gmail.com; 4 Department of Recreation and Leisure Industry Management, National Taiwan Sport University, Taoyuan 33301, Taiwan

**Keywords:** strength and conditional training, power exercise training, sake protein, anti-fatigue, exercise performance

## Abstract

Exercise and fitness training programs have attracted the public’s attention in recent years. Sports nutrition supplementation is an important issue in the global sports market. Purpose: In this study, we designed a power exercise training (PET) program with a mouse model based on a strength and conditional training protocol for humans. We tested the effect of supplementation with functional branched-chain amino acid (BCAA)-rich sake protein (SP) to determine whether the supplement had a synergistic effect during PET and enhanced athletic performance and resistance to fatigue. Methods: Male ICR mice were divided into three groups (*n* = 8 per group) for four-week treatment: sedentary controls with vehicle (SC), and PET and PET groups with SP supplementation (3.8 g/kg, PET + SP). Exercise performance was evaluated by forelimb grip strength and exhaustive swimming time as well as changes in body composition and anti-fatigue activity levels of serum lactate, ammonia, glucose, and creatine kinase (CK) after a 15-min swimming exercise. The biochemical parameters were measured at the end of the experiment. Results: four-week PET significantly increased grip strength and exhaustive swimming time and decreased epididymal fat pad (EFP) weight and area. Levels of aspartate aminotransferase (AST), alanine aminotransferase (ALT), creatinine, and uric acid (UA) were significantly increased. PET + SP supplementation significantly decreased serum lactate, ammonia and CK levels after the 15-min swimming exercise. The resting serum levels of AST, ALT, CREA and UA were all significantly decreased with PET + SP. Conclusion: The PET program could increase the exercise performance and modulate the body composition of mice. PET with SP conferred better anti-fatigue activity, improved biochemical profiles, and may be an effective ergogenic aid in strength training.

## 1. Introduction

Sake is a Japanese-style alcohol beverage; it results from fermenting rice that has been polished to remove the bran. The bran is a byproduct from the sake industry, known as “Sake Lees” or “Sake protein (SP)”. SP is abundant in protein, carbohydrates and amino acids. It is one kind of plant protein-based nutritional supplement. Many recent studies investigated whey protein (milk protein-base; WP) marketed as a dietary supplement and as an aid for muscle development with resistance training [1,2]. Because of its rapid rate of digestion, WP provides a rapid source of amino acids that can be taken up by the muscles to repair and rebuild muscular tissue. However, no study has investigated whether a plant protein-based nutritional supplement, SP, can affect muscle development, exercise performance and anti-fatigue activity. SP is enriched in branched-chain amino acids (BCAAs) such as valine, isoleucine and leucine, and glutamic acid. Leucine turns on the translational machinery necessary for muscle protein synthesis and has been shown to activate the mammalian target of rapamycin (mTOR) signaling pathway [3,4]. Resistance exercise has a potent and acute effect on mTOR signaling and muscle protein synthesis [5,6].

The benefits of traditional anaerobic resistance training are widely established and accepted by health professionals. Core strength and conditioning training have been used widely in recent years and include CrossFit training [7], strength and conditioning training [8], and high-intensity interval training [9]. These training programs are unique in their focus on maximizing neuroendocrine response, developing power, cross-training with multiple training modalities, constant training and practice with functional movements and the development of successful diet strategies [10]. The National Strength and Conditioning Association (NSCA) workout design focuses on maximum muscular strength, maximum muscular power, muscle endurance, anaerobic capacity, aerobic capacity, agility, speed, body composition, flexibility, and anthropometry [11]. The CrossFit domains are similar to those defined by the NSCA, with 11 domains of fitness: cardiovascular, respiratory endurance, stamina, strength, flexibility, power, speed, coordination, agility, balance and accuracy [12].

Research is lacking on SP supplementation combined with NSCA- or CrossFit-like training for body composition, serum biochemical indexes, exercise performance and anti-fatigue activity. We designed a strength training protocol for mice based on an NSCA- and CrossFit-like training program to simulate these exercises in an animal model. We aimed to investigate the beneficial synergistic effects of SP supplementation and power exercise training (PET) on exercise performance, biochemical profiles, and pathological responses after long-term supplementation. SP supplementation may be helpful to athletes focusing on strength and condition-based training for maximal strength performance and muscle endurance to enhance muscle maximal power and improve exercise capability as well as for overall physiologic protective effects.

## 2. Experimental Section

### 2.1. Materials, Animals, and Experiment Design

SP used for supplementation in the study was obtained from Taiwan Tobacco & Liquor Corporation (TTL), Minister of Finance, Executive Yuan, Republic of China. The nutrition facts and total hydrolyzed amino acids of SP were analyzed by SGS Taiwan, Ltd. (New Taipei City, Taiwan) and are shown in Table 1. Male ICR strain mice (6 weeks old) with specific pathogen-free conditions were purchased from BioLASCO (A Charles River Licensee Corp., Yi-Lan, Taiwan). One week of acclimation to the environment and diet was allowed before the experiment began. All animals were provided a standard laboratory diet (No. 5001; PMI Nutrition International, Brentwood, MO, USA) and distilled water *ad libitum*, and housed at 12-h light/12-h dark cycle at room temperature (24 ± 1 °C) and 50%–60% humidity. The Institutional Animal Care and Use Committee (IACUC) of National Taiwan Sport University inspected all animal experiments in this study, and the study conformed to the guidelines of protocol IACUC-10323 approved by the IACUC ethics committee.

All animals were randomly assigned to 3 groups (8 mice/group) for PET and SP supplementation administered by oral gavage: (1) sedentary control with vehicle (SC); (2) PET received vehicle; (3) PET with SP supplementation (PET + SP). The same volume of solution relative to individual body weight was received by the SC, PET and PET + SP groups.

### 2.2. SP Supplementation

Mice in the PET + SP group were given SP within 30 min after the PET. The recommended use of SP for humans is about 18.5 g per one intake with a normal diet and exercise program. The mouse SP dose (3.8 g/kg) used in this study was converted from a human equivalent dose on the basis of body surface area by the following formula from the US Food and Drug Administration [13]: assuming a human weight of 60 kg, the human equivalent dose of 18.5 g/60 kg (0.308 g/kg) = 0.308 × 12.3 = a mouse dose of 3.8 g/kg; the conversion coefficient 12.3 was used to account for differences in body surface area between a mouse and a human.

### 2.3. PET Protocol

**Figure 1:**
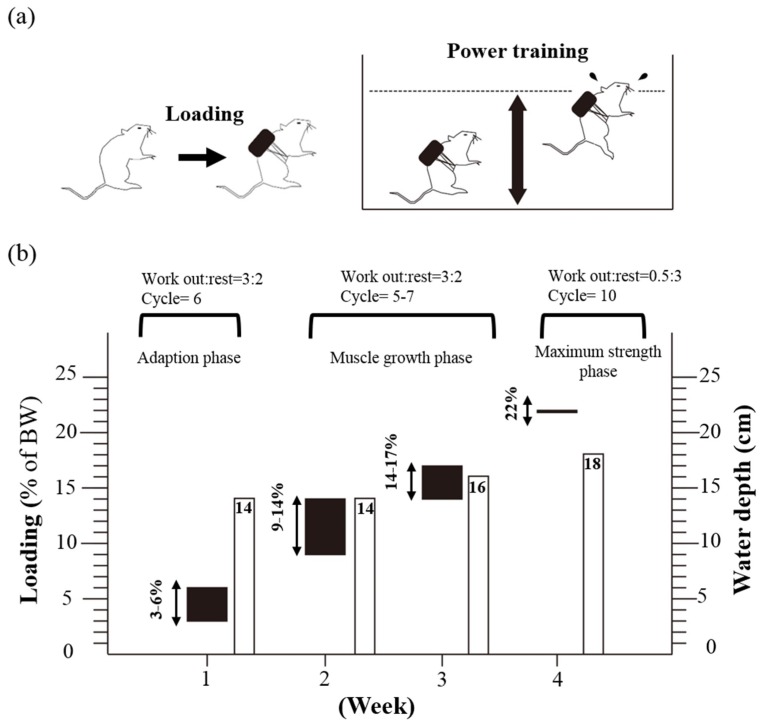
Protocol for 4-week power exercise training (PET). (**a**) Mice were placed in a plastic container (65 cm high, 20 cm in diameter) with tap water 14–18 cm deep maintained at 30 ± 1 °C; (**b**) The training program of four-week PET.

Animals in the PET and PET + SP groups underwent PET following the training protocol shown in Figure 1. The PET program was based on an NSCA-like training program and modified from other studies [14,15,16,17]. Mice were placed in a plastic container (65 cm high, 20 cm in diameter) with tap water 14–18 cm deep maintained at 30 ^o^C ± 1 ^o^C. The strength training involved 3 periods: adaption phase, muscle growth phase and maximum muscle strength phase. At the first week, mice were trained using a protocol: rest (3 min: 2 min) for 6 cycles of forced swimming (14 cm deep) with a 3% to 6% loading of body weight for the adaption phase. The muscle growth phase was during the second and third week; mice trained for workout: rest (1 min: 1 min) for 5–7 cycles of forced swimming (14 cm deep) with a 10% to 14% body-weight loading. The third week, the loading was increased to 14% to 17% body weight for the forced swimming (16-cm deep water) for workout: rest (1 min: 1 min) for 5 cycles. A load of 22% body weight was defined as the maximum muscle strength phase; mice trained for workout: rest (0.5 min: 3 min) for 10 cycles of forced swimming (18-cm deep water). The frequency of the training protocol was 5 times a week.

### 2.4. Forelimb Grip Strength

A low-force testing system (Model-RX-5, Aikoh Engineering, Nagoya, Japan) was used to measure the forelimb grip strength of mice. The amount of tensile force was measured by use of a force transducer equipped with a metal bar (2 mm diameter and 7.5 cm long). The detailed procedures were described in our previous studies [18,19]. Forelimb grip strength was tested after consecutive administration of control, PET and PET + SP treatment for 4 weeks and 1 h after the last treatment. The maximal force (in grams) recorded by this low-force system was used as the grip strength.

### 2.5. Swimming Exercise Performance Test

Mice were pretreated with the control, PET and PET + SP for four weeks, then underwent an exhaustive swimming test that began 1 h after the last treatment administration. The details of the endurance swimming test were described previously [20,21]. The endurance of each mouse was recorded as the time from the beginning to exhaustion, determined by observing loss of coordinated movements and failure to return to the surface within 7 s. Times floating, struggling, and making necessary movements were considered in the swimming duration until exhaustion and possible drowning. In order to avoid drowning, exhaustive time of each swimming mouse was recorded in 2 of 3 of the following: loss of coordinated movements, failure to return to the surface within 7 s, and lose the swimming ability.

### 2.6. Determination of Blood Biochemical Variables

The effect of PET and PET + SP on serum lactate, ammonia, and glucose levels and CK activity were evaluated post-exercise. At 1 h after the last administration, a 15-min swimming test was performed without weight loading, then blood samples were immediately collected from the submandibular duct of pretreated mice and centrifuged at 1500 ×*g* and 4 °C for 10 min for serum preparation. Lactate, ammonia, and glucose levels and CK activity in serum were determined by using an autoanalyzer (Hitachi 7060, Hitachi, Tokyo). The other biochemical variables, shown in Table 2, were measured by using Beckman DxC 800 analyzer (Beckman Coulter, Brea, CA, USA).

### 2.7. Tissue Glycogen Determination

Liver and muscle tissues were investigated to determine whether PET and PET + SP treatment increased glycogen deposition. About 1 h after the last treatment administration, mice were sacrificed by CO_2_ inhalation. The liver was excised and weighed. The method of glycogen analysis was described in our previous studies [22,23].

### 2.8. Histological Staining of Tissues and Calculated Epididymal Fat Pad Area

Different tissues were collected and fixed in 10% formalin after mice was sacrificed. After the formalin fixed, the epididymal fat pad (EFP) was put on graph paper and calculated the area (cm^2^). Sections were cut transversely or longitudinally to obtain ventricular sections or four-chamber cross-sections, respectively. Tissues were then embedded in paraffin and cut into 4-μm thick slices for morphological and pathological evaluations. Tissue sections were stained with hematoxylin and eosin (H & E) and examined by light microscopy with a CCD camera (BX-51, Olympus, Tokyo, Japan) by a clinical pathologist.

### 2.9. Statistical Analysis

All data are expressed as mean ± SD. Statistical differences among groups were analyzed by a one-way analysis of variance (ANOVA) with use of SAS 9.0 (SAS Inst., Cary, NC, USA). *p* < 0.05 was considered statistically significant.

## 3. Results and Discussion

### 3.1. Nutrition Facts and Total BCAAs of SP

Nutritional facts and total BCAAs of SP supplement are in Table 1. The SP was rich in protein, with less sugar and fat. The BCAA content of valine, isoleucine and leucine was 1.73, 1.30 and 2.25 g/100g, respectively. Thus, SP had high protein and BCAA content, which could increase protein synthesis in skeletal muscle and have anabolic effects on protein metabolism by increasing the rate of protein synthesis and decreasing the rate of protein degradation in resting human skeletal muscle [24].

### 3.2. Effect of SP Supplementation and PET on Body Weight (BW), Skeletal Muscle Mass, and Other Metabolism-Related Organ Weights

**Figure 2:**
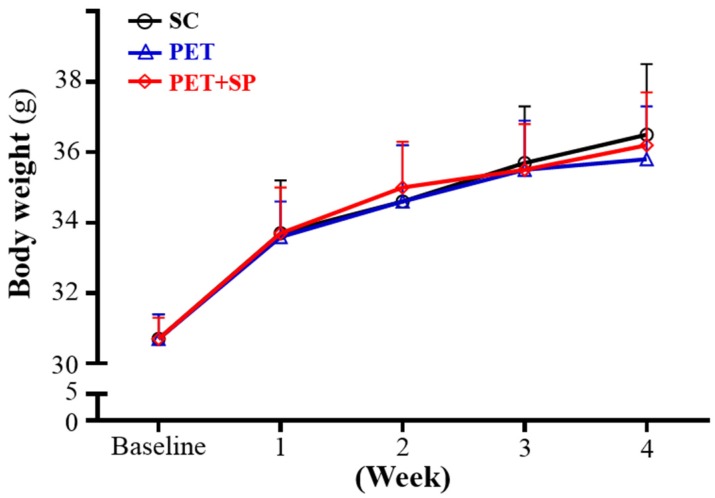
Increased body weight in mouse groups with sedentary control with vehicle (SC), PET and PET + sake protein supplementation (PET + SP) mice for four weeks. Data are mean ± SD for *n* = 8 mice per group.

The initial BW of SC, PET and PET + SP groups was 30.7 ± 0.7, 30.7 ± 0.7 and 30.7 ± 0.6 g, respectively, with no differences between groups (Figure 2). The BW regardless of PET or PET + SP treatment did not differ from the controls during the training and SP supplementation. Thus, our PET program and SP supplementation did not affect BW. In previous studies, some training programs could decrease BW because the participants or animals were obese or patients had metabolic syndrome [25,26]. In general, a medium- or long-term exercise training program could not change the BW greatly. BW change is complicated and is related to mechanistic energy imbalance including changes in resting metabolic rate, non-exercise activity, fat-free mass and energy intake [27].

**Figure 3:**
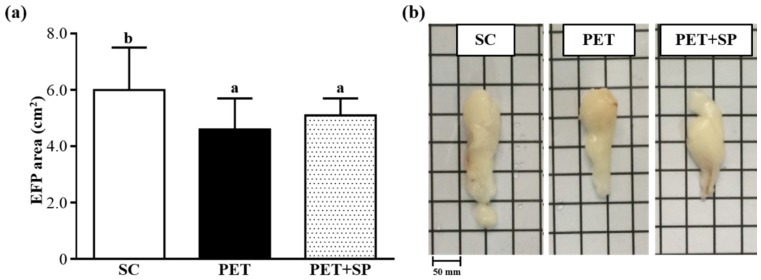
Effect of SP supplementation and four-week PET on (**a**) epididymal fat pad (EFP) area (cm^2^). (**b**) View of EFP. Data are mean ± SD of eight mice in each group by one-way ANOVA. Different letters (a, b) indicate a significant difference at *p* < 0.05.

The morphological data from each experimental group are summarized in Table 2. The food and water intake of the treated groups did not differ. In addition, there were no significant changes in the liver, kidney, heart, lung and muscle weight. The EFP weight for PET and PET + SP groups in the average values was significantly lower, by 31.6% (*p* = 0.0034) and 32.8% (*p* = 0.0025), respectively, compared to the control group. The brown adipose tissue (BAT) weight was significantly higher for the PET than SC group, by 1.13-fold (*p* = 0.0326). Moreover, relative tissue weight (%) is a measure of different tissue weights adjusted for individual BW. The relative EFP weight of the PET and PET+SP groups in the average values was 31.0% (*p* = 0.0042) and 33.5% (*p* = 0.0024) lower than SC group, respectively. Relative liver, kidney, heart, lung, muscle and BAT weight did not differ among groups. We also calculated EFP area (Figure 3a) and observed EFP morphology (Figure 3b). The EFP area for SC, PET and PET + SP groups was 6.0 ± 1.5, 4.6 ± 1.1 and 5.1 ± 0.6 cm^2^, respectively. Compared with the SC group, PET and PET + SP groups showed decreased EFP area in the average values, by 22.56% (*p* = 0.0023) and 15.67% (*p* = 0.0128), respectively. Consistent with the change in BW and tissue weights, the PET program had no negative impact on appetite, BW or tissue weight but transformed the body composition of the adipose tissue, increasing brown-like fat, which is supplied with energy sources to build muscle, and decreasing white fat tissue [28]. Furthermore, SP supplementation had no adverse effects on food and water intake, BW and tissue weight during the PET program period.

### 3.3. Effect of SP Supplementation with PET Program on Forelimb Grip Strength

**Figure 4:**
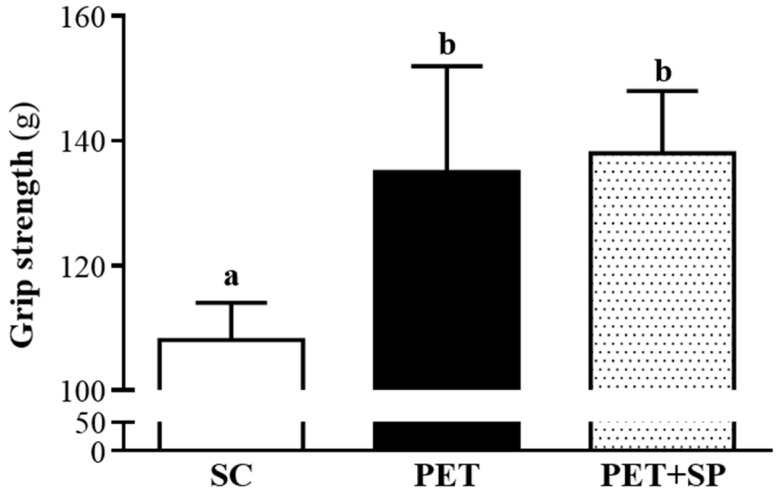
Effect of SP supplementation and four-week PET on forelimb grip strength. Male ICR mice underwent a grip strength test 1 h after the final administered dose. Data are mean ± SD of eight mice in each group by one-way ANOVA. Different letters (a, b) indicate a significant difference at *p* < 0.05.

Data for grip strength were 108 ± 6, 135 ± 17 and 138 ± 10 g for the SC, PET and PET + SP groups, respectively (Figure 4), and was significantly higher with PET training (*p* = 0.0002) and PET+SP (*p* < 0.0001), respectively, than with SC treatment. Thus, PET could improve grip strength; however, PET+SP supplementation could not further improve forelimb grip strength. The forelimb grip demonstrates the maximal and explosive force production. In our previous study, ICR mice with swimming endurance training also showed increased grip strength [29]. However, our previous training program was an endurance training for six weeks and without an interval. Therefore, two different types of training programs could improve grip strength, but we could not judge which training program is more efficient to improve the explosive force production, which needs further study.

### 3.4. Effect of SP Supplementation with the PET Program on Exercise Performance in a Weight-Loaded Swimming Test

**Figure 5:**
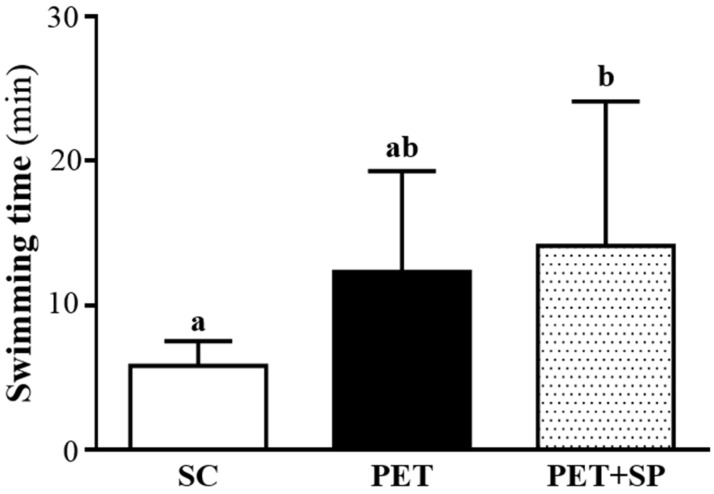
Effect of SP supplementation and four-week PET on swimming exercise performance. Mice were pretreated with SP and then 1 h later performed an exhaustive swimming exercise with a load equivalent to 5% of the mouse’s body weight attached to the tail. Data are mean ± SD of eight mice in each group by one-way ANOVA. Different letters (a, b) indicate a significant difference at *p* < 0.05.

Exercise endurance is an important variable in evaluating aerobic capacity. In our study, the exercise endurance levels with a swimming test in mice administered SC, PET and PET + SP were 5.8 ± 1.7, 12.3 ± 7.0 and 14.1 ± 10 min, respectively (Figure 5). The swimming time was significantly longer (*p* = 0.0287) with PET + SP than SC treatment, with no significant difference between SC and PET alone groups. SP supplementation could enhance endurance capacity under the PET program and benefited endurance exercise performance under training intervention conditions but (as was seen from the grip strength experiment) there no effect on explosive force production could be seen.

### 3.5. Effect of SP Supplementation under the PET Program on Serum Lactate, Ammonia, Glucose, CK and BUN Levels after Acute Exercise Challenge

**Figure 6:**
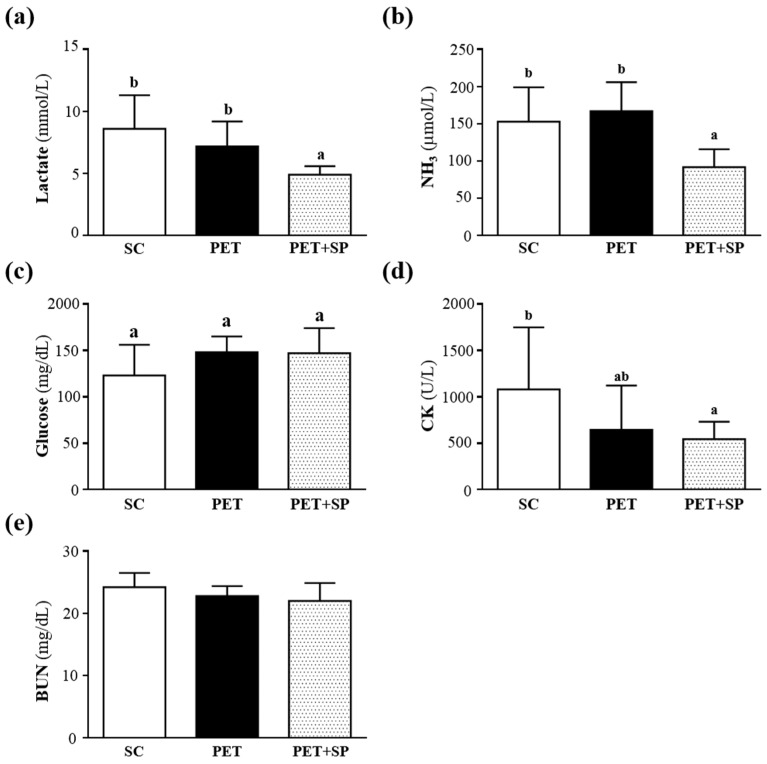
Effect of SP supplementation and four-week PET on serum levels of (**a**) lactate, (**b**) ammonia, (**c**) glucose, (**d**) creatine kinase (CK), and (**e**) blood urea nitrogen (BUN) after an acute exercise challenge. Mice were pretreated with of SP for four weeks, then 1 h later performed a 15-min swimming test without weight loading. Data are mean ± SD of eight mice in each group. Columns with different letters (a, b) significantly differ at *p* < 0.05 by a one-way ANOVA.

Muscle fatigue after exercise can be evaluated by biochemical indicators including lactate, ammonia, glucose, CK and BUN [30]. During high-intensity exercise, muscles must obtain sufficient energy from anaerobic glycolysis, and abundant lactate is produced by glycolysis metabolism. Lactate is an oxidizable substrate in skeletal muscle and a precursor to gluconeogenesis in muscles or liver after exercise [31]. In the present study, lactate levels in the SC, PET and PET + SP groups were 8.6 ± 2.7, 7.2 ± 2.0 and 4.9 ± 0.7 mmol/L; the level with PET + SP treatment was significantly lower in the average values, by 43.3% (*p* = 0.0012) and 31.9% (*p* = 0.0289), than with SC and PET treatment, respectively (Figure 6a).

Muscle fatigue is associated with deamination of adenine nucleotides, and increased deamination of AMP coincides with decreased phosphocreatine and pH values and failure of the contraction process. Peripheral and central fatigue levels are related to increased ammonia level during exercise [32]. Serum ammonia levels in the control, PET and PET + SP groups were 153 ± 46, 167 ± 39 and 92 ± 24 μmol/L, respectively. Values for the PET + SP group were significantly lower in the average values, by 19.87% (*p* = 0.0034) and 44.91% (*p* < 0.0001) than the control and PET alone groups, respectively (Figure 4b). Glucose, a breakdown product of tissue glycogen, is released as a circulating substrate for energy utilization after intense exercise [33]. During exercise, carbohydrates are the main substrates for ATP resynthesis in tissues, and glucose mobilization is associated with the metabolic demands of muscles during activity [34]. The maintenance of steady levels of blood glucose during physical exercise involves very precise controls of the hepatic production of glucose, which includes hormonal feedback mechanisms [35]. Therefore, blood glucose levels are an important index for performance maintenance during exercise. Serum glucose levels in the SC, PET and PET + SP groups were 123 ± 33, 148 ± 17 and 147 ± 27 mg/dL, respectively; with no statistically significant differences among groups (Figure 4c).

Serum CK is an important clinical biomarker for muscle damage, such as muscular dystrophy, severe muscle breakdown, myocardial infarction, autoimmune myositides, and acute renal failure. CK activity in the SC, PET and PET + SP groups was 1081 ± 668, 644 ± 478 and 544 ± 188 U/L, respectively (Figure 3d), and PET+SP group (*p* = 0.0386) was significantly lower than the SC group (Figure 4d). Therefore, SP supplementation should ameliorate skeletal muscle injury induced by acute exercise challenge. Serum BUN (blood urea nitrogen), creatinine, and urine output were closely monitored to measure renal function. Many factors other than renal disease can cause BUN alteration [36]. Urea is formed by the liver and carried with the blood to the kidneys, and urea is an important index correlation with protein breakdown, dehydration, stress and fatigue [37]. Level of serum BUN in the SC, PET and PET + SP groups was 24.2 ± 2.3, 22.8 ± 1.6 and 22 ± 2.9 mg/dL, respectively, with no statistically significant difference among groups (Figure 4e). Therefore, SP could be used under the PET program to reduce serum lactate, ammonia and CK levels after acute exercise challenge and shows anti-fatigue activity during the PET period. Above all, SP supplementation may be an ergogenic supplement to recover the fatigue and recovery of muscle damage under the high-intensity training program.

### 3.6. Effect of PET and PET+SP on Hepatic and Muscle Glycogen Level

**Figure 7:**
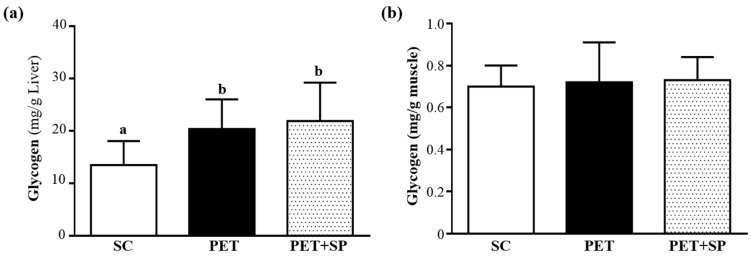
Effect of SP supplementation and four-week PET on (**a**) hepatic glycogen and (**b**) muscle glycogen levels at rest. All mice were sacrificed and examined for glycogen levels of muscle and liver tissues 1 h after the final treatment. Data are mean ± SD of eight mice in each group.

With energy expenditure during exercise, physical fatigue is mainly caused by energy consumption and deficiency [38]. Glycogen is the predominant source of glycolysis [39]. During exercise, serum glucose can be derived from three possible sources: diet (although only if one eats during exercise), liver glycogenolysis and hepatic gluconeogenesis. Thus, glycogen stores have been considered one of the most important factors limiting the maintenance of moderate to high power output for extended periods of time [40]. Therefore, glycogen storage directly affects exercise ability. We measured glycogen content of liver and muscle tissues (Figure 7a, b). The liver glycogen levels in SC, PET and PET + SP groups were 13.48 ± 4.56, 20.35 ± 5.68 and 21.87 ± 7.36 mg/g liver, respectively; the liver glycogen content with PET (*p* = 0.0317) and PET + SP treatment (*p* = 0.0105) were significantly higher than that with SC treatment (Figure 7a). Leucine can modulate glucose uptake by strong involvement in the protein translation initiation pathway, which is related to glucose homeostasis by GLUT4 translocation to the sarcolemma. Thus, SP can modulate glucose metabolism via insulin-dependent and -independent pathways [41]. Glycogen content of muscle tissues did not differ among groups (Figure 7b). Muscle glycogen is important fuel for ATP synthesis, and storage at rest is a critical factor for maintaining physical performance during endurance exercise [42]. Previous study suggested that when carbohydrates are available after exercise, liver glycogen resynthesis is the first priority and muscle glycogen synthesis is secondary [43]. According to our data, although supplementation with SP under the PET program did not increase hepatic and muscle glycogen storage and synthesis, our PET program could increase glycogen synthase and maintain the muscle glycogen levels to retain enhance strength and energy utilization.

### 3.7. Effect of PET and PET+SP on Biochemical Analyses at the End of the Experiment

We observed beneficial effects of SP on the exhaustive exercise challenge and strength enhance and measured other physiological effects with four-week SP supplementation and a PET program. In Table 3, levels of biochemical factors, including glucose, CK, BUN, ALB and total protein (TP), did not differ among groups. However, levels of creatinine, AST, ALT and UA in the average values were 1.32- (*p* = 0.0008), 1.66- (*p* = 0.0027), 1.55- (*p* = 0.0045) and 1.33-fold (*p* = 0.0180), respectively, higher in the PET than SC group. In addition, creatinine and ALT levels in the average values with PET+SP treatment were 28.6% (*p* = 0.0002) and 37.9% (*p* = 0.0026), respectively, significantly lower than with SP treatment. The levels of serum AST and UA were slightly increased with PET + SP treatment and did not differ from the SC group. Thus, our PET program could slightly induce oxidative stress with creatinine, AST, ALT, and UA activities. SP supplementation during the PET period could ameliorate the high-intensity exercise training inducing oxidative stress. The biochemistry of urea, creatinine, and UA can reflect renal damage. During renal ischemia-reperfusion injury, the burst of reactive oxygen species, mainly produced by xanthine oxidase, can trigger inflammation and tubular cell injury and subsequent generation of UA [44]. AST and ALT levels indicate oxidative impact, as evidenced by the significant increase in hepatic lipid peroxidation and cell damage markers [45]. SP supplementation may have potential applications for liver and renal protection under high-intensity training according to our *in vivo* data.

### 3.8. Histopathological Evaluation of PET and PET + SP Treatments at the End of the Experiment

**Figure 8:**
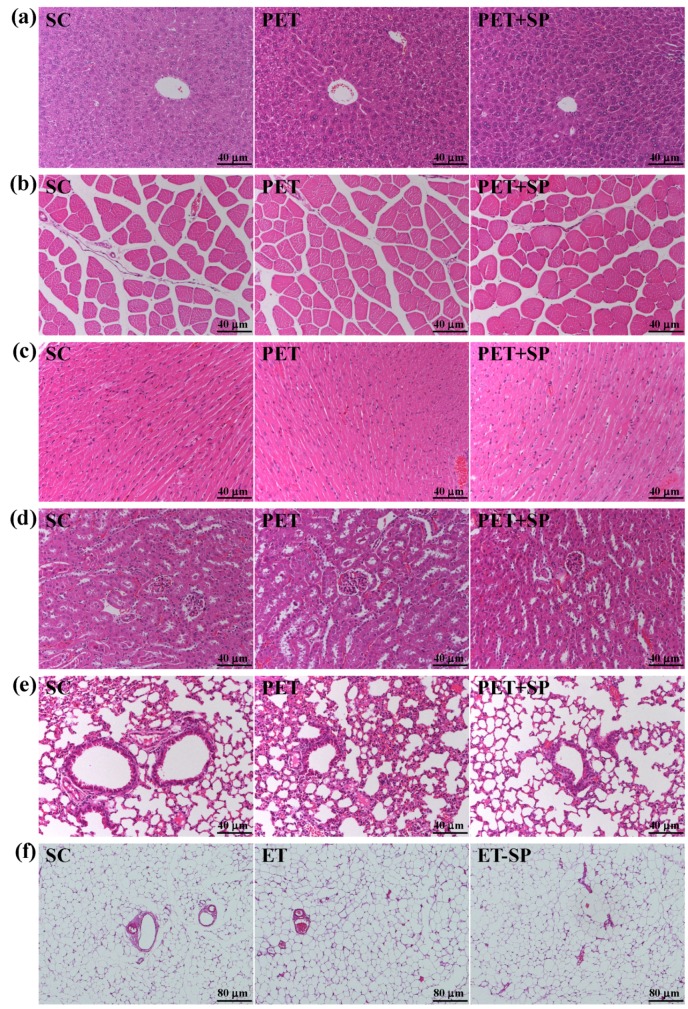
Effect of PET and PET + SP on the morphology of (**a**) liver, (**b**) skeletal muscle, (**c**) heart, (**d**) kidney, (**e**) lung and (**f**) epididymal fat pad (EFP) tissues. Specimens were photographed with a light microscope (Olympus BX51). (H & E stain, magnification: a-e, ×200, Scale bar, 40 µm; f, ×100, Scale bar, 80 µm).

Our PET and PET+SP treatments had no toxic effects on major organs such as the liver, skeletal muscles, heart, or kidney, lung and adipose according to histopathological examinations (Figure 8). Lung slices from our PET group showed an increase in alveolar macrophages and slight inflammation, and the PET + SP group showed larger and fewer alveoli and decreased inflammation as compared with PET alone and controls (Figure 8e). In previous study, the high accumulation of blood markers of metabolic stress observed in a post-training exercise protocol [46] were similar to our data. SP supplementation may ameliorate the PET program inducing metabolic and oxidative stress.

## 4. Conclusions

In this study, our SP supplement was abundant in protein, carbohydrate and hydrolyzed amino acids, particularly BCAAs: valine, isoleucine and leucine content reached 5.28 g/100 g. Glucose is an important fuel for exercising muscles; the scarcity of carbohydrate reserves in liver reduces pyruvate levels that are a substrate for both acetyl-coenzyme A formation and inception of anaplerotic reaction for continuous oxidation of free fatty acids or amino acids [47]. However, valine and isoleucine cannot be completely converted into glucose in the liver, but they could be utilized in the muscles [48]. In our study, we found that a four-week PET program significantly decreased white adipose tissue and EFP weight and increased BAT weight. These finding suggested that NSCA- or CrossFit-like training could decrease body fat and change body composition. As well, physical exercise performance increased in both endurance and explosive force capacity under the PET program. Although SP supplementation did not improve body composition or exercise performance under our PET program, values for exercise-induced fatigue-related variables were improved. In addition, SP supplementation had beneficial effects on the liver and renal and lung functions under the high-intensity training program. SP may be a potential ergogenic used in a strength and conditioning training-based program to reduce fatigue caused by training and may be favorable for biochemical variables. We also provide basic safety evidence from pathological observations and assessments. This study suggests alternative uses of SP as a nutrient supplement with potential positive health impacts, and the effect of SP treatment on NSCA- or CrossFit-like training programs in humans requires further study.

## Figures and Tables

**Table 1:** Nutrition facts, hydrolyzed amino acid profiles and total branched-chain amino acids of sake protein (SP).

Nutrition Facts	Content
Nutrition Facts	/100 g SP
Protein	30.2 g
Fat	2.8 g
Saturated fat	1.36 g
Trans fat	0
Carbohydrate	58.3 g
Sugar	4 g
Moisture	7.9 g
Ash	0.8 g
Sodium	11.8 mg
Total calories	379.2 Kcal
Hydrolyzed amino acid profiles	g/100g
Serine	1.32
Aspartic Acid	2.07
Glutamic Acid	3.95
Glycine	1.26
Histidine	0.67
Arginine	1.90
Threonine	1.12
Alanine	1.53
Proline	1.22
Cystine	0.59
Tyrosine	1.22
Phenylalanine	1.39
Methionine	0.63
Lysine	0.99
Tryptophan	0.37
Valine	1.73
Isoleucine	1.30
Leucine	2.25

Nutrition and hydrolyzed amino acid profiles were analyzed by SGS Taiwan Ltd.

**Table 2:** General characteristics of the experimental groups.

Characteristic	SC	PET	PET + SP	*p*-value between PET & SC	*p*-value between PET + SP & SC
Initial BW (g)	30.7 ± 0.7	30.7 ± 0.7	30.7 ± 0.6	0.8594	0.9435
Final BW (g)	37.1 ± 2.1	36.8 ± 1.2	37.6 ±1.4	0.7197	0.5039
Food intake (g/day)	6.3 ± 0.5	6.2 ± 0.8	6.3 ± 0.8	0.5911	0.8673
Water intake (mL/day)	7.9 ± 1.5	7.6 ± 1.0	7.9 ± 1.3	0.3929	0.9326
*Weight (g)*					
Liver	1.88 ± 0.16	1.87 ± 0.06	1.89 ± 0.09	0.7745	0.9297
Kidney	0.58 ± 0.04	0.58 ± 0.03	0.58 ± 0.03	0.1000	0.9407
Heart	0.26 ± 0.04	0.25 ± 0.05	0.26 ± 0.04	0.6924	0.8210
Lung	0.25 ± 0.02	0.24 ± 0.02	0.24 ± 0.02	0.3043	0.3673
Muscle	0.39 ± 0.02	0.39 ± 0.02	0.39 ± 0.02	0.9183	0.6823
EFP	0.50 ± 0.11 ^b^	0.34 ± 0.11 ^a^	0.34 ± 0.06 ^a^	0.0034	0.0025
BAT	0.123 ± 0.014 ^a^	0.139 ± 0.016 ^b^	0.135 ± 0.012 ^ab^	0.0326	0.0930
*Relative Weight (%)*					
Liver	5.08 ± 0.41	5.08 ± 0.23	5.02 ± 0.25	0.9741	0.6799
Kidney	1.57 ± 0.12	1.58 ± 0.11	1.54 ± 0.07	0.8485	0.5678
Heart	0.69 ± 0.11	0.67 ± 0.15	0.69 ± 0.10	0.7614	0.9838
Lung	0.67 ± 0.07	0.65 ± 0.04	0.64 ± 0.06	0.4179	0.2288
Muscle	1.05 ± 0.07	1.06 ± 0.10	1.04 ± 0.03	0.8129	0.8657
EFP	1.35 ± 0.29 ^b^	0.93 ± 0.29 ^a^	0.90 ± 0.19 ^a^	0.0042	0.0024
BAT	0.33 ± 0.04 ^a^	0.38 ± 0.04 ^b^	0.36 ± 0.04 ^a^	0.0578	0.2373

Data are mean ± SD for *n* = 8 mice in each group. Values in the same line with different superscripts letters (a, b) differ significantly, *p* < 0.05 by one-way ANOVA. Muscle mass includes both gastrocnemius and soleus muscles in the back part of the lower legs. BW: body weight; BAT: brown adipose tissue; EFP: epididymal fat pad; sedentary control with vehicle (SC), power exercise training (PET) and PET + sake protein supplementation (PET + SP).

**Table 3:** Effect of SP supplementation and 4-week PET on biochemical serum levels at the end of the experiment.

Variable	SC	PET	PET+SP	*p*-value between PET & SC	*p*-value between PET+SP & SC
AST (U/L)	94 ± 37 ^a^	156 ± 46 ^b^	125 ± 22 ^ab^	0.0027	0.1063
ALT (U/L)	62 ± 9 ^a^	95 ± 34 ^b^	59 ± 10 ^a^	0.0045	0.8160
CK (U/L)	413 ± 149	418 ± 205	412 ± 114	0.9582	0.9840
Albumin (g/dL)	3.81 ± 0.20	3.90 ± 0.32	3.90 ± 0.21	0.4910	0.4910
TP (g/dL)	5.2 ± 0.2	5.2 ± 0.2	5.2 ± 0.2	1.0000	0.7999
BUN (mg/dL)	21.1 ± 2.4	21.1 ± 3.3	21.5 ± 1.6	0.9922	0.7481
Creatinine (mg/dL)	0.27 ± 0.04 ^a^	0.35 ± 0.05 ^b^	0.25 ± 0.04 ^a^	0.0008	0.5291
UA (mg/dL)	1.4 ± 0.2 ^a^	1.8 ± 0.4 ^b^	1.7 ± 0.4 ^ab^	0.0180	0.0892
Glucose (mg/dL)	146 ± 13	143 ± 16	143 ± 16	0.6976	0.7216

All mice were sacrificed at the end of the experiment and examined for serum clinical biochemistry variables. Data are mean ± SD for *n* = 8 mice per group. Values in the same line with different superscripts letters (a–c) differ significantly, *p* < 0.05 by one-way ANOVA. AST, aspartate aminotransferase; ALT, alanine aminotransferase; CK, creatine kinase; TP, total protein; BUN, blood urea nitrogen; UA, uric acid.
